# Group membership and adolescents’ third-party punishment: a moderated chain mediation model

**DOI:** 10.3389/fpsyg.2023.1251276

**Published:** 2023-12-11

**Authors:** Zhen Zhang, Menghui Li, Qiyun Liu, Chao Chen, Chunhui Qi

**Affiliations:** ^1^Faculty of Education, Henan Normal University, Xinxiang, China; ^2^Faculty of Education, Henan University, Kaifeng, China; ^3^Mental Health Education Center, Nanyang Medical College, Nanyang, China; ^4^Zhumadian Basic Teaching Research Office, Zhumadian, China

**Keywords:** group membership, third-party punishment, unfair perception, anger, adolescents

## Abstract

Third-party punishment (TPP) reflects people’s social preference for fairness norms and is fundamental to maintaining fairness norms on a large scale. Several empirical studies have shown that the offender’s group membership impacts TPP, but the detailed mechanisms have yet to be fully elucidated. The current study used the third-party punishment game task to explore the relationship between group membership, perceived unfairness, anger, and adolescents’ TPP. A total of 306 teenagers aged 12 to 15 were chosen as subjects through cluster sampling. The results showed that group membership (classmate vs. stranger) and gender can affect adolescents’ TPP together, which manifests as adolescents enacting significantly harsher punishments on strangers than on classmates, especially for boys. Group membership indirectly affects TPP through the mediating effects of perceived unfairness, anger and through a chain mediation of perceived unfairness and anger. Moreover, gender positively moderate the relationship between group membership and perceived unfairness. Specifically, group membership significantly affects boys’ perceived unfairness, but cannot predict girls’ perceived unfairness. The above results can be used to guide adolescents toward appropriate justice concepts and moral awareness, thus enhancing TPP.

## 1 Introduction

As an important way to safeguard social fairness, third-party punishment (TPP) refers to behavior in which individuals voluntarily provide resources to punish violators in response to irregularities (Fehr and Gächter, 2002). Behavioral economists and evolutionary psychologists emphasize that TPP can effectively suppress potential non-cooperative behavior, which is not only beneficial to the establishment and maintenance of long-term relationships but also helps to promote and maintain stability and harmony in society (Buckholtz and Marois, 2012). Scholars often use the third-party punishment game (TPPG) to explore how individuals deal with violations that do not involve their own interests and the factors that impact them. During this task, unrelated third-party participants observed an individual (i.e., a transgressor) providing an unfair distribution to a recipient (i.e., give $2 out of $10 to the recipient and keep $8 for yourself), and then decided whether to punish the selfish transgressor at their expenses (Fehr and Fischbacher, 2004). People across diverse societies have a willingness to punish unfair players (Henrich et al., 2006; House et al., 2020), and this behavior is crucial for maintaining social cooperation (Balliet et al., 2014; Henrich and Muthukrishna, 2021).

The importance of TPP has attracted attention in many disciplines due to its role in promoting group cooperation and maintaining social order (Krueger and Hoffman, 2016; Marshall and McAuliffe, 2022). To understand the origin and development of third-party’ fairness consideration, several studies have examined the TPP of children at different developmental stages (Gummerum and Chu, 2014; McAuliffe et al., 2015; Gummerum et al., 2016, 2020, 2022; Lee and Warneken, 2022). Six-year-old children begin to exhibit costly TPP (McAuliffe et al., 2015; Riedl et al., 2015; Salali et al., 2015), and the punishment pattern fully develops until 13–14 years of age (Bašić et al., 2020) and has a certain cross-cultural stability (House et al., 2020). However, to date, most related studies have examined children and adults as third-party punishers, and few studies have examined adolescents (Gummerum et al., 2020, 2022). Adolescence, defined as the period from 10 to 24 years of age (Sawyer et al., 2018), is characterized by heightened affective and social sensitivity (Towner et al., 2023). Moreover, group influence is highly prevalent during adolescence, which made adolescence more concerned with conformity and fitting in with others (Blakemore, 2018). Accordingly, adolescents may show exhibit more intense TPP than children and adults. Ultimately, there is a need to explore the factors that influence TPP among juveniles.

### 1.1 Group membership and TPP

Group membership is a social dimension that distinguishes oneself from others, including friendship, race, class, nationality, and even mere membership triggered by artificial cues (Lieberman and Linke, 2007; Chierchia et al., 2020; Zhang et al., 2021, 2022). Researchers have examined the effect of group membership on TPP, but their findings have been inconsistent. Two competing hypotheses, the *Mere Preferences Hypothesis* and the *Norms Focused Hypothesis* (McAuliffe and Dunham, 2016; Zhang et al., 2020), were developed to explain the contradictory results. The *Mere Preferences Hypothesis* suggests that individuals’ positive evaluation toward the ingroup would reduce TPP for ingroup perpetrators, supported by the majority of evidence based on adults (Yudkin et al., 2016; McAuliffe et al., 2017; Guo et al., 2020, 2022; Yang et al., 2023) and children (Jordan et al., 2014), which supports the ingroup favoritism phenomenon (IGF). The *Norms Focused Hypothesis* emphasizes that individuals’ concern for maintaining norms of group cooperation would enhance TPP for ingroup violators, as demonstrated by some evidence based on adults (Mendoza et al., 2014; Delton and Krasnow, 2017) and children (Gonzalez-Gadea et al., 2022), known as the black sheep effect (BSE). Even though they differ in the direction of the effect, IGF and BSE are two ways for people to maintain the group identity and cohesion (Zhang et al., 2020). However, various systematic reviews and meta-analyses have found that children, adolescents, and adults are more likely to punish outgroup offenders than ingroup criminals (McAuliffe and Dunham, 2016; Lazić et al., 2021). Therefore, group membership can influence adolescents’ TPP, showing that youth punish outgroup members more harshly than in-group members (Hypothesis 1).

### 1.2 Perceived unfairness as a potential mediator

Perceived unfairness is one potential explanation for the proposed effect of group membership on TPP (Lu and McKeown, 2018). Fairness preference theory suggests that humans have a strong disgust for inequality and are willing to consume resources to punish offenders when they experience or witness injustices (Fehr and Schmidt, 1999). Some studies have shown that adolescents have strong equity concerns and a high willingness to sacrifice their personal interests to uphold fairness norms; thus, perceived unfairness has become an important driving force for implementing punishment (Güth and Kocher, 2014; Lu and McKeown, 2018). Nevertheless, the perception of injustice is not invariable, and it will depend on the group relationship of both sides. Firstly, individuals’ perception of injustice is less prominent when unfair proposals are made by ingroups than by outgroups (Lu and McKeown, 2018). In addition, perceived unfairness is associated with TPP, such that the greater the perceived unfairness, the more motivated people are to punish (Fehr and Gächter, 2002). Finally, self-reported justice perception mediates the relationship between partners’ social distance (i.e., human vs. computer partner) and rejection behavior among healthy adults and patients with major depressive disorders (Wang and Li, 2013; Jin et al., 2022). Therefore, we proposed that group membership may be associated with more TPP for outgroup members via increased perceived unfairness (Hypothesis 2).

### 1.3 Anger as a potential mediator

According to negative emotion theory, perceiving negative emotions such as anger, frustration, and disgust that arise from behavior violations can form the basis for punishing behaviors, triggering a desire to punish others in response to real-life immorality (Hartsough et al., 2020). Self-reported anger has been suggested as a possible motivation for TPP in some studies (Fehr and Fischbacher, 2004; Gummerum et al., 2016) and could mediate the association between unfair offers and TPP in adults (Gummerum et al., 2020). Additionally, the harshness of TPP increased significantly when anger was induced but decreased when anger was inhibited (Gummerum et al., 2022). More importantly, the experience of anger caused by injustice differs depending on the peer group to which one belongs. Bicskei et al. (2016) found that the same unkind behavior by outgroups was associated with greater anger-like emotions than that of ingroups, and punishment behavior was strongly influenced by anger-like emotions. Finally, Wang and Li (2013) found that self-reported feelings of anger could mediate the association between social relations (i.e., friend, teacher, and stranger) and adults’ punishment in the ultimatum game. Hence, we proposed that anger played a crucial role in the relationship between group membership and TPP (Hypothesis 3).

### 1.4 Perceived unfairness and anger

An evaluation-emotional-behavioral model was employed by Seip et al. (2014) to explain the mechanism underlying costly punishments of unfairness: evaluating an action or event as unjust leads to anger toward the offender, which can then drive people to punish those who violate social norms, even if punishment comes at a price. Several studies have indicated that violations of fairness can lead to perceived unfairness, which leads to anger and, ultimately motivates punishment by second and third parties (Singer and Steinbeis, 2009; Mendoza et al., 2014). For example, Mendoza et al. (2014) demonstrated that increasingly unfair offers predicted lower perceived fairness, thereby resulting in a strengthened level of anger and ultimately prompting individuals to reject the unfair offers in ultimatum game. Accordingly, we proposed that perceived unfairness and anger can exert a chain-mediating effect between group membership and TPP (Hypothesis 4).

### 1.5 Gender as a potential moderator

Social role theory emphasizes that different societal stereotypes are assigned to boys and men as compared to girls and women, with girls expected to be more communal and caring and boys are expected to be agentic and dominant (Eagly, 2009). These gender role beliefs greatly influence boys’ and girls’ perceptions, emotional experiences, and behavioral responses to norm-violating behavior (Chawla et al., 2020). Laboratory and field studies suggest that boys are more likely to judge private behavior negatively, experience greater anger, and punish offenders more severely when they experience normal transgressions than girls (Kromer and Bahçekapili, 2010; Bonini et al., 2011; Balafoutas and Nikiforakis, 2012; Rodriguez-Ruiz et al., 2019). As a result of this socialization and other forces, boys and men tend to be more socially dominant than girls and women (Pratto et al., 1994; Du et al., 2021), and norms for masculinity are more rigid than norms for femininity (Koenig, 2018). Accordingly, boys show greater ingroup favoritism both cognitively, emotionally and behaviorally than girls during intergroup interactions. For example, boys exhibit greater ingroup favoritism than girls when responding to unfair distributions (Wu and Gao, 2018). Thus, we proposed that the chain mediation of perceived unfairness and anger was more pronounced in boys than in girls (Hypothesis 5).

## 2 Method

### 2.1 Participants and procedure

The current study adopted a complete between-subject design of 2 (group membership: classmate vs. stranger) × 2 (gender: boy, girl). Based on an *a priori* power analysis, the sample size was estimated using G*Power 3.1 (Faul et al., 2007). F tests and ANOVA (fixed effects, special effects, main effects, and interactions) in G*Power (version 3.1.9.7) were selected. To detect a medium effect (f^2^ = 0.25), *N* = 128 participants (32 participants per group) with 0.80 power and 0.05 Types I error rates were needed. Experimental data were collected from two junior high schools in Henan Province, China. The distribution and collection of situational questionnaires were conducted by a trained research assistant with standardized processes for completing the questionnaires. During the study, eight classes of seventh- and eighth-graders were randomly selected. Four classes were randomly assigned to the classmate condition, and the other four classes were assigned to the stranger condition.

A total of 350 questionnaires were distributed in the form of class tests. After removing missing values or other ineffective responses, the final data set consisted of 306 questionnaires, with a minimum of 49 respondents for each condition. The sample included 175 boy students (57.19%) and 131 girl students (42.81%) between the ages of 12 and 15. Their average age was 13.46 ± 0.75 years, with 69.99% in seventh grade and 33.01% in eighth grade. All subjects self-reported no mental or psychological disorders and gave their oral informed consent. Ethics committee approval was obtained from the Faculty of Education at Henan Normal University, and protocol adherence to the Declaration of Helsinki was ensured.

### 2.2 Experimental procedure and materials

Students were instructed to complete a pen-and-paper test on TPP in the classroom as a class. There were four main sections of the assessment, including basic personal information, third-party punishment tasks, group membership manipulation and check, and self-report assessment. These four sections were always administered in the same order as below.

#### 2.2.1 Third-party punishment game

Based on the third-party punishment game paradigm designed by Fehr and Fischbacher (2004), a situational questionnaire was developed and administered as follows:

To celebrate the National Day of China, your school held a literary and artistic performance. Two students, Li Ming and Wang Hua, collaborated in singing “I and My Motherland” and won first place in the competition. The school awarded a cash award of 100 (RMB) to the winning team. Li Ming, as a representative, went on stage to receive the award. The judge teacher reminded Li Ming that Wang Hua also contributed to this award and asked the two of them to share the award, allowing Li Ming to decide on how to allocate the money. Li Ming then provided an allocation scheme of 80:20, which means that Li Ming received 80 RMB, while Wang Hua received 20 RMB.

#### 2.2.2 Group membership manipulation and check

In accordance with a previous study (Guo et al., 2016), we manipulate group membership by asking participants to imagine that the offender (Li Ming) is a classmate of theirs (ingroup condition) vs. is from a different class (outgroup condition). In both cases, the third-party victim (Wang Hua) was depicted as a stranger, both to the participants and to the offender. The Inclusion of Other in the Self (IOS) scale developed by Aron et al. (1992) was used to assess the perceived social distance between the two parties, and then the effectiveness of group membership manipulation was tested. The scale mainly uses the size of the overlapping area of two circles to determine the degree of closeness between the two circles, ranging from a complete distance of 1 point to an approximate overlap of 5 points. This article uses the Likert 5-point scoring method; the higher the score, the higher the degree of social distance.

#### 2.2.3 Self-report assessment

Participants were told that imagined themselves as bystanders in the above scenario and assessed the following three aspects: (1) unfairness perception, that is, the unfairness degree of the allocation scheme of 80:20, measured on a scale of 1 to 7, with higher scores representing higher perceived unfairness (Lu and McKeown, 2018); (2) anger, that is, how angry you feel about the unfair allocation, measured on a scale of 1 to 7, with higher scores representing more anger (Gummerum et al., 2020); and (3) punishment intensity, that is, the amount of punishment the participants are willing to impose, measured on a scale from 0 to 4, with a punishment ratio of 1:20 (each punishment amount will reduce the offender by 20 yuan) (Chen and Bo, 2016).

## 3 Results

### 3.1 Manipulation check

**Figure 1:**
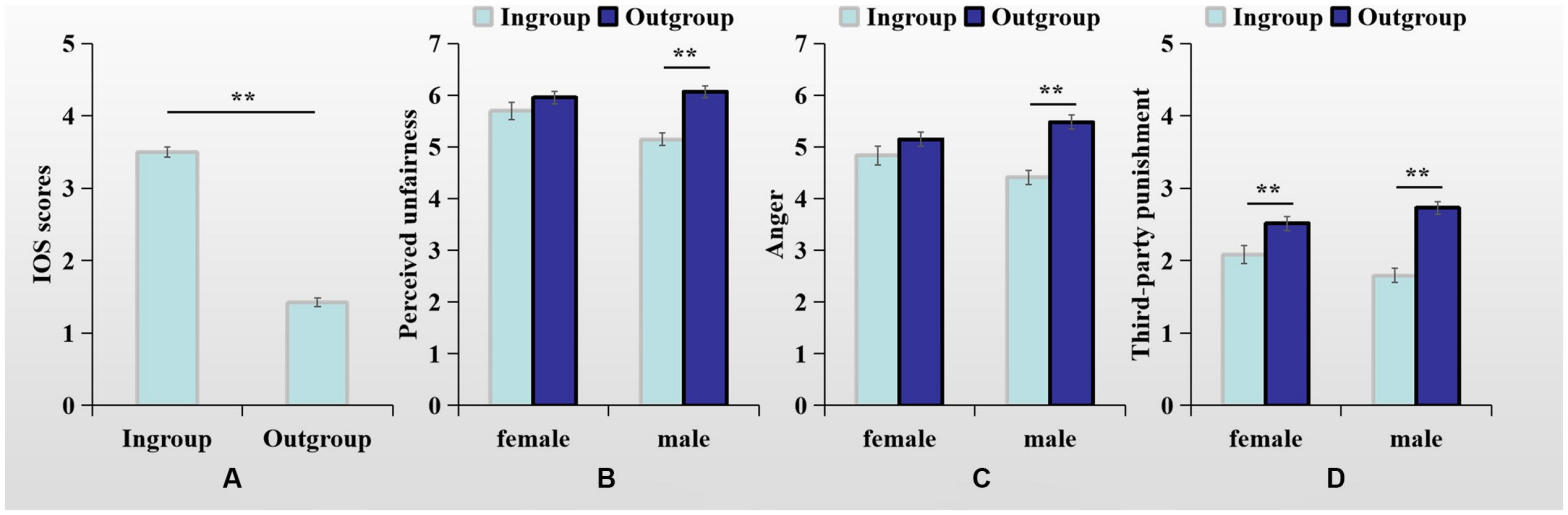
**(A)** IOS scores as a function of group membership; **(B)** Perceived unfairness, **(C)** anger and **(D)** third-party punishment as a function of group membership, separately for girls and boys. Error bars indicate standard error. *p < 0.05, **p < 0.01.

A 2 (group membership: classmate vs. stranger) × 2 (gender: boy vs. girl) ANOVA on the IOS scale scores showed that only the main effect of group membership was significant, *F*(1,302) = 588.52, *p* < 0.01, partial η^2^ = 0.66. Identification with a classmate was larger (*M* = 3.50, *SE* = 0.07) than identification with stranger (*M* = 1.42, *SE* = 0.06), see Figure 1A. This finding indicated that the manipulation of group membership was successful.

### 3.2 Preliminary analyses

A 2 (group membership: classmate vs. stranger) × 2 (gender: boy vs. girl) MANOVA on perceived unfairness, anger, and TPP found that the main effects of group membership were significant, *F*s(1,302) > 19.75, *p*s < 0.01, partial η^2^s > 0.06. As compared to strangers’ selfish behavior, participants perceived unfair allocation from classmates as less unfair, experienced less anger, and punished less severely. Moreover, the interactions by group membership and gender were also significant, *F*s(1,302) > 6.13, *p*s < 0.05, partial η^2^s > 0.02. Further analysis shown that girls’ TPP was influenced by group membership, *F*(1,302) = 7.67, *p* < 0.01, but not by their perceptions of unfairness and anger, *F*s(1,302) < 1.82, *p*s > 0.05. In particular, girls punished classmates (*M* = 2.08, *SE* = 0.12) less severely than stranger (*M* = 2.51, *SE* = 0.10). In contrast, boys’ perceptions of unfairness, anger, and TPP were affected by group membership, *F*s(1,302) > 29.23, *p*s < 0.01, showing that boys perceive classmate’ transgressions as less unfair, experience less anger, and impose softer punishments comparing to stranger’s transgressions (see Figures 1B–D). The main effects of gender were not significant, *F*s(1,302) < 2.69, *p*s > 0.05.

The descriptive statistics and correlations of the variables are reported in Table 1. Dummy codes were used for group membership, with ingroup coded as 0 and outgroup coded as 1. Group membership was significantly positively associated with perceived unfairness, anger, and TPP (*r* = 0.28, 0.28, 0.38, *p*s < 0.01), supporting Hypothesis 1. Unfair perception was significantly positively associated with anger and TPP (*r* = 0.68, 0.67, *p*s < 0.01). Anger was significantly positively correlated with TPP (*r* = 0.67, *p* < 0.01).

**Table 1:** Descriptive statistic and correlations of variables (*N* = 306).

Variable	*M*	SD	1	2	3	4	5
1. Gender	0.57	0.50	–	–	–	–	–
2. Group membership	0.57	0.50	−0.10	–	–	–	–
3. Perceived unfairness	5.73	1.18	−0.10	0.28**	–	–	–
4. Anger	5.00	1.33	−0.02	0.28**	0.68**	–	–
5. Third-party punishment	2.31	0.94	−0.04	0.38**	0.67**	0.67**	–

Gender and group membership is a virtual variable, female and ingroup = 0, male and outgroup = 1; *p < 0.05, **p < 0.01.

### 3.3 Moderated chain mediation model

A moderated chain mediation model was conducted by using Model 85 in the Process 4.0 macro of SPSS 26.0. Dummy codes were used for gender and group membership, with girl and ingroup coded as 0 while boy and outgroup coded as 1. Confounding effects were reduced by including age and grade as control variables. The results showed that group membership could significantly positively predict unfair perception, anger and TPP (*β* = 0.27, 0.09, 0.18, *p*s < 0.01); unfair perception could significantly positively predict anger and TPP (*β* = 0.67, 0.36, *p*s < 0.01); and anger could significantly positively predict TPP (*β* = 0.37, *p* < 0.01). Thus, Hypothesis 2, 3 and 4 were supported. The interaction between group membership and gender had a significant effect on perceived unfairness (*β* = 0.14, *p* < 0.05). In contrast, the interaction between group membership and gender had no effect on anger (*β* = 0.04, *p* > 0.05) and TPP (*β* = 0.03, *p* > 0.05). Thus, Hypothesis 5 was partially supported (see Table 2).

**Table 2:** The moderated chain mediating effect of perceived unfairness and anger.

Regression equation		Overall fitting index			Regression coefficient	
Result variable	Prediction variable	*R*	*R* ^2^	*F*	*β*	*t*
Perceived unfairness	Group membership	0.33	0.11	7.44**	0.27	4.90**
	Gender				−0.08	−1.48
	Group membership × Gender				0.14	2.56*
Anger	Perceived unfairness	0.71	0.50	50.08**	0.67	15.39**
	Group membership				0.09	2.17*
	Gender				0.05	1.28
	Group membership × Gender				0.04	1.03
Third-party punishment	Anger	0.75	0.56	54.08**	0.37	6.83**
	Perceived unfairness				0.36	6.54**
	Group membership				0.18	4.55**
	Gender				0.03	0.77
	Group membership × Gender				0.03	0.78

All variables in the model are brought back into the equation after standardized processing. Gender and group membership is a virtual variable, female and ingroup = 0, male and outgroup = 1; *p < 0.05, **p < 0.01.

**Figure 2:**
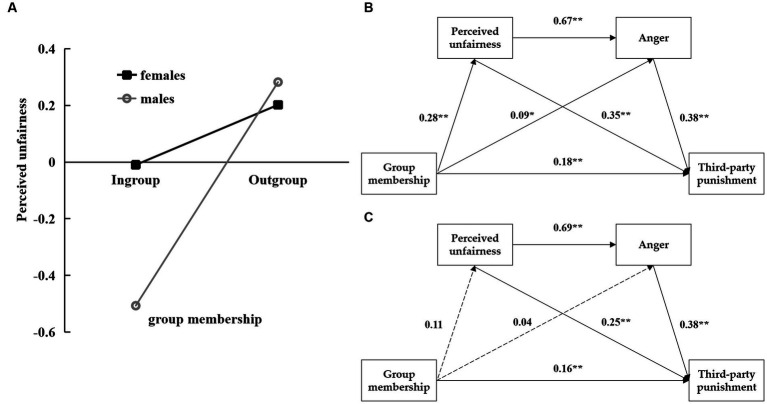
The moderating role of gender in the relation between group membership and perceived unfairness **(A)**; The figures of the chain mediation model separately for boys **(B)** and girls **(C)**.

A slope test was conducted to clarify the mechanisms by which group membership and gender interact with perceived unfairness. The result showed that group membership significantly positively predict the boys’ perceived unfairness (simple slope = 0.39, *t* = 5.47, *p* < 0.01), but cannot predict girls’ perceived unfairness (simple slope = 0.11, *t* = 1.23, *p* > 0.05) (see Figure 2A). The figures of the chain mediation model separately for boys and girls were shown in Figures 2B,C.

## 4 Discussion

The current study explores the relationship between group membership and adolescent’s TPP and its potential mechanism. The findings show that group membership and gender could affect adolescents’ perceived unfairness, anger, and TPP together, which manifests as boys perceive classmates’ transgressions as less unfair, experience less anger, and impose softer punishments compared to strangers’ transgressions. Furthermore, group membership weakens adolescent’s TPP through perceived unfairness, anger and a chain mediating path of perceived unfairness and anger, especially for boys.

Our results support the *Mere Preferences Hypothesis*, because adolescents’ perceived unfairness, anger, and TPP both exhibited IFG instead of BSE. These findings are aligned with previous research based on adults (Yudkin et al., 2016; McAuliffe et al., 2017; Guo et al., 2020, 2022; Yang et al., 2023) and children (Jordan et al., 2014), which indicated that people are more likely to forgive ingroup offenders than outgroup offenders. From the perspective of psychological development, the replicated IFG effect in adolescents not only extends previous studies, but also coincides with recent meta-analysis results (Lazić et al., 2021). In other words, adolescents, like children and adults, care about and defend their group membership and are willing to forgive in-group violators. However, Gonzalez-Gadea et al. (2022) found that children aged 6 to 9 exhibited an ingroup policing bias but not an ingroup favoritism bias. One potential explanation for the difference is the cost of punishment. Yudkin et al. (2019) found that costly punishment, as a more effective way of group regulation, produces ingroup policing effects, rather than non-costly punishment. The TPP decision used in our study involves costless self-reported punishment, which might lead to IGF instead of BSE.

Moreover, perceived unfairness mediates the relationship between group membership and TPP for junior school students. In particular, outgroup infractions are perceived as more unjust in comparison to ingroup violations, thereby promoting TPP. Consistent with previous research (McCall et al., 2014; Lu and McKeown, 2018), the perception of injustice is comparatively less pronounced when inequitable propositions originate from ingroup as opposed to outgroup, regardless of whether the resource allocation scenario involves second or third parties. Based on the *Mere Preferences Hypothesis* (McAuliffe and Dunham, 2016), the identity of groups may lead to a positive appraisal and partiality toward ingroups, thereby fostering greater inclusivity toward ingroup offenders. Brain imaging research has suggested that individuals utilize mentalizing networks to comprehend and justify transgressions committed by ingroup members, which subsequently leads to weaker perceived unfairness (Baumgartner et al., 2012; Fatfouta et al., 2018). Furthermore, this aligns with prior studies that have demonstrated the role of perceived injustice as a mediator in the association between social distance and retribution enacted by a second party (Wang and Li, 2013; Jin et al., 2022). Thus, in comparison to classmates, third-party bystanders tend to view transgressions committed by strangers as more unjust, which subsequently results in severe TPP.

Once more, the relationship between group membership and adolescents’ TPP is mediated by anger. Specifically, strangers’ infraction triggers stronger anger than classmates’ infraction, leading to more severe punishment. This also supports the *Mere Preferences Hypothesis*, showing that ingroup violations are emotionally tolerated by people (McAuliffe and Dunham, 2016). As previously demonstrated (McCall et al., 2014; Bicskei et al., 2016), anger emotions were less salient when unfair allocations were provided by ingroups than outgroups. Moreover, this finding is in agreement with Wang and Li (2013), finding that anger mediated the link between social relations and rejection during ultimatum game. Thereby, strangers’ violations cause third-party bystanders to feel more angry than classmates, resulting in harsher TPP.

In addition, perceived unfairness and anger can serve as a chain-mediating mechanism linking group membership and adolescents’ TPP. The evaluation-emotional-behavioral model suggests that evaluating an event as unjust leads to anger toward the offender, which may then lead to punishment for violating social norms, even if punishment is costly (Seip et al., 2014). These results imply that ingroup violations induced stronger perceived unfairness than outgroup violations, resulting in a reduced level of anger and ultimately prompting individuals to exhibit a lower TPP. Our findings are consistent with previous research, finding that perceived unfairness and anger exerted a chain-mediating effect between fairness consideration and second-party punishment (Singer and Steinbeis, 2009; Mendoza et al., 2014). Consequently, identification with a classmate can influence an individual’s perception and evaluation of an unfair event, subsequently impacting the level of anger experienced and ultimately altering the degree of TPP.

Finally, as previously reported among children (Wu and Gao, 2018), preliminary results indicated that boys perceive classmates’ violation as less unfair, experience less anger, and impose softer punishments compared to strangers’ violations, while girls only exhibit a small IGF on TPP. When gender was incorporated into the model, gender could negatively moderate the relationship between group membership and perceived unfairness. It supports the social role theory that boys have stronger IGF than girls (Eagly, 2009). This gender difference may be caused by different societal stereotypes and socialization processes for boys and girls (Rose and Rudolph, 2006). In adolescence, social norms expect boys’ prescriptive roles to be agent, dominant, and assertive, while girls’ prescriptive roles to be warm, communal and supportive (Koenig, 2018). Consequently, boys have stronger IGF than girls as a result of these experiences.

## 5 Implications of the study

To our knowledge, our research is the first to demonstrate IGF among adolescents’ TPP. This finding has significant implications for the broader question of how morality is formed and developed. First, our results indicate that TPP is biased from childhood through adolescence and into adulthood, which completes the developmental trajectories associated with this bias. Second, an individual’s perceived unfairness, anger and chain mediation between them may be a psychological mechanism contributing to this bias. Third, the indirect path of group membership and perceived unfairness is significant for boys, but not for girls, implying that gender modulates this indirect path. Using these results, we can better understand when and how group biases develop and who is more likely to exhibit them.

## 6 Limitations and future research

Like previous research, this study is subject to several limitations. Initially, the third-party punishment game used in our study involves costless self-reported punishment, which might be different from incentivized punishment (Gummerum et al., 2016, 2020, 2022; Gonzalez-Gadea et al., 2022). Future studies should explore how TPP with real monetary incentives are affected by group membership. Furthermore, the identity of the classmate was not controlled. Different participants may have imagined different types of classmates and this could substantially increase the variance of classmate’s IOS. This differentiation might substantially affect adolescents’ interpersonal decision-making (Burnett Heyes et al., 2015), which needs to be strictly controlled in future studies. Finally, it is worth considering that various factors, such as compassion and social orientation value, could influence the association between group membership and TPP. Therefore, future research endeavors could benefit from the inclusion of additional variables in order to gain a more comprehensive understanding of this relationship.

## 7 Conclusion

Our results indicated that adolescents enacted more severe sanctions to stranger’s violation than to classmate’s violation during the third-party punishment task. Moreover, perceived unfairness and anger had a chain-mediating effect on the relationship between group membership and TPP. Additionally, the indirect path of group membership and perceived unfairness is significant for boys, but not for girls. These findings contribute to a deeper comprehension of the development mechanism of group bias in adolescents’ TPP.

## Data availability statement

The raw data supporting the conclusions of this article will be made available by the authors, without undue reservation.

## Ethics statement

The studies involving humans were approved by the Faculty of Education at Henan Normal University. The studies were conducted in accordance with the local legislation and institutional requirements. Written informed consent for participation in this study was provided by the participants’ legal guardians/next of kin.

## Author contributions

CQ and ZZ designed the experiment. ML, QL, and CC collected and analyzed the data. ML and ZZ wrote the manuscript. All authors contributed to the article and approved the submitted version.
